# No effect of gestational diabetes or pre-gestational obesity on 6-year offspring left ventricular function—RADIEL study follow-up

**DOI:** 10.1007/s00592-020-01571-z

**Published:** 2020-07-28

**Authors:** Linda Litwin, Johnny K. M. Sundholm, Kristiina Rönö, Saila B. Koivusalo, Johan G. Eriksson, Taisto Sarkola

**Affiliations:** 1 Children’s Hospital, University of Helsinki and Helsinki University Hospital, Helsinki, Finland; 2 Department of Congenital Heart Defects and Pediatric Cardiology, FMS in Zabrze, Medical University of Silesia, Katowice, Poland; 3 Women’s Hospital, University of Helsinki and Helsinki University Hospital, Helsinki, Finland; 4 University of Helsinki and Helsinki University Hospital, Helsinki, Finland; 5 Folkhälsan Research Center, Helsinki, Finland; 6 Yong Loo Lin School of Medicine, National University of Singapore, Singapore, Singapore; 7 Minerva Foundation Institute for Medical Research, Helsinki, Finland

**Keywords:** Developmental biology, Gestational diabetes mellitus, Obesity, Child, Cardiovascular disease, Ventricular function

## Abstract

**Aims:**

We aimed to investigate associations between pre-pregnancy obesity, gestational diabetes (GDM), offspring body composition, and left ventricular diastolic and systolic function in early childhood.

**Methods:**

This is an observational study, including 201 mother–child pairs originating from the Finnish Gestational Diabetes Prevention Study (RADIEL; 96 with GDM, 128 with pre-pregnancy obesity) with follow-up from gestation to 6-year postpartum. Follow-up included dyads anthropometrics, body composition, blood pressure, and child left ventricular function with comprehensive echocardiography (conventional and strain imaging).

**Results:**

Offspring left ventricular diastolic and systolic function was not associated with gestational glucose concentrations, GDM, or pregravida obesity. Child body fat percentage correlated with maternal pre-pregnancy BMI in the setting of maternal obesity (*r* = 0.23, *P* = 0.009). After adjusting for child lean body mass, age, sex, systolic BP, resting HR, maternal lean body mass, pre-gestational BMI, and GDM status, child left atrial volume increased by 0.3 ml (95% CI 0.1, 0.5) for each 1% increase in child body fat percentage.

**Conclusions:**

No evidence of foetal cardiac programming related to GDM or maternal pre-pregnancy obesity was observed in early childhood. Maternal pre-pregnancy obesity is associated with early weight gain. Child adiposity in early childhood is independently associated with increased left atrial volume, but its implications for long-term left ventricle diastolic function and cardiovascular health remain unknown.

**Electronic supplementary material:**

The online version of this article (10.1007/s00592-020-01571-z) contains supplementary material, which is available to authorized users.

## Introduction

Maternal hyperglycaemia affects foetal development and results in increased newborn morbidity, including macrosomia, left ventricle (LV) hypertrophy, and diastolic dysfunction even in optimally treated pregnancies [1–3]. Maternal obesity and gestational diabetes mellitus (GDM) are associated with an early offspring weight gain and predict increased adiposity and unfavourable cardiometabolic risk profiles later in life [4, 5]. Long-term cardiovascular health has a multifactorial background, and an independent effect of an altered intrauterine development has been hypothesized. Biological mechanisms remain the matter of ongoing research, and foetal programming related to maternal obesity and GDM has been frequently suggested [6].

Ventricular hypertrophy is known to disturb myocardial relaxation, but LV diastolic dysfunction in the setting of GDM has been observed even in normal ventricles [7]. GDM-related LV diastolic dysfunction is usually benign and transient in the clinical setting with typical remission in early infancy. Apart from foetal development, LV myocardial relaxation can be affected by child growth and health behaviours, including the obesity-related functional decline observed in adolescents [8]. Whether unfavourable intrauterine cardiovascular development attributed to maternal obesity and GDM could manifest as myocardial dysfunction later in life is largely unknown. The lack of evidence limits our ability to predict offspring long-term cardiovascular risk with respect to prenatal history.

In our previous work, we have found that maternal pregravida obesity, but not GDM was associated with offspring early vascular structure changes, but neither had the effect on child LV mass in early childhood [9, 10]. In this study, we aim to investigate associations between maternal pre-pregnancy obesity, GDM, offspring body composition, and LV diastolic and systolic function.

## Material and methods

The Supplementary Material for this article includes details of body composition and echocardiographic assessment (Online Resource 1).

### Study design

This longitudinal observational follow-up study included 201 mother–child pairs originating from the population of Finnish Gestational Diabetes Prevention Study (RADIEL; *N* = 728 women). We aimed for a 50% prevalence of GDM in our subcohort. Follow-up assessment at 6.1 years (± 0.5) postpartum was accomplished between June 2015 and May 2017 and included child echocardiography, child and maternal anthropometrics, body composition, blood pressure (BP), blood glucose and lipids, vascular ultrasound, and tonometry. The first evaluation was designed in 5–6-year postpartum to ensure child cooperation without sedation. The Ethics Board of Helsinki University Hospital approved the study. Informed written consent was obtained from all of the mothers.

RADIEL is a randomized controlled multicentre interventional trial in women with increased risk of GDM (history of GDM or pre-pregnancy body mass index (BMI) exceeding 30 kg/m^2^) [11]. Women planning a pregnancy or in the first half of gestation were randomized into the intervention (counselling on diet and physical activity) or a control (standard care) arm. Maternal gestational data were collected prospectively. GDM was diagnosed in reference to concurrent guidelines (at least one pathological glucose value in a 75 g two-hour oral glucose tolerance test during gestation—fasting ≥ 5.3 mmol/l, one-hour ≥ 10.0 mmol/l, two-hour ≥ 8.6 mmol/l) [12]. If glucose values repeatedly exceeded 5.5 mmol/l before breakfast or 7.8 mmol/l one hour after a meal, metformin or insulin treatment was started. In our subcohort, child characteristics and studied outcomes did not differ with respect to the intervention (*N* = 97, 48%) and we opted not to analyse this further due to the potential selection bias.

### Anthropometrics

Follow-up height and weight were measured with electronic devices (Seca GmbH & Co. KG, Germany) to the nearest 0.1 cm and 0.1 kg. Child BMI *Z*-scores were generated in reference to the recent Finnish population dataset [13]. Body surface area was calculated with Haycock formula. Maternal pre-pregnancy BMI was based on measured or declared weight and measured height.

### Body composition

Child lean body mass was measured by bioelectrical impedance (InBody 720, InBody Bldg, Korea) and calculated with the previously validated formula (based on age, sex, height, weight, and BMI *Z*-score) [14], with strong correlation (*r* = 0.951). Cardiovascular follow-up was separated from a bioelectrical impedance visit by a median of 1.0 years (range 0.05–2.32). To avoid the bias, we rely on equation predicting body composition, generated from body size data during imaging. Body fat percentage was calculated as (weight–lean body mass)/weight. Lean body mass measured with InBody 720 was reported for mothers.

### BP and samples

Resting BP was measured in the sitting position from the right arm with adequate cuffs with oscillometry (Omron M6W, Omron Healthcare Europe B.V., The Netherlands). Mean systolic and diastolic BPs were calculated from the two lowest measurements (out of a minimum three). Child BPs *Z*-scores were calculated in reference to the guidelines [15]. Plasma glucose was assessed with enzymatic assays and glycated haemoglobin A_1c_ (HbA_1c_) with immunoturbidimetric analyser (Roche Diagnostics, Switzerland).

### Echocardiography

**Fig. 1:**
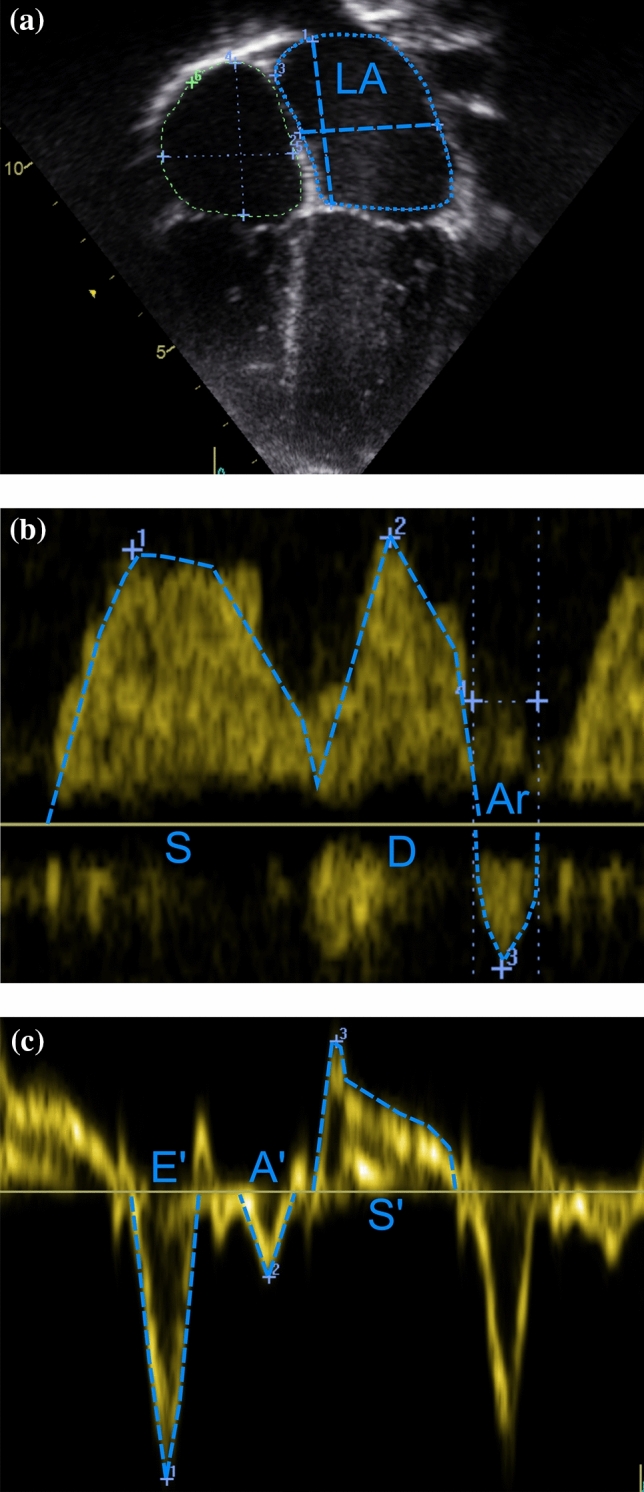
Assessment of left ventricular diastolic function: **a** Left atrial volume calculation with biplane area-length method (apical four-chamber view at ventricular end-systole showing left atrial major-axis length and left atrial planimetered area). **b** Pulmonary venous systolic-to-diastolic peak velocity ratio (S/D), pulmonary venous A wave reversal (Ar) amplitude and duration with Doppler. **c** Mitral lateral peak early and late diastolic, and systolic tissue velocities (E′, A′, S′) with Tissue Doppler Imaging.

Comprehensive child echocardiography was performed by one experienced paediatric cardiologist (TS) and analysed by one experienced observer (LL) blinded to maternal and child characteristics. Images were obtained and measurements performed according to guidelines [16, 17]. LV diastolic function was assessed integrating left atrial volume, Doppler, Tissue Doppler, and strain imaging (Fig. 1). LV systolic function was evaluated with conventional and strain imaging. The results were normalized for body surface area and converted to *Z*-scores using recent high-quality paediatric data obtained with the same echocardiography protocol, matched for child race and age [18–20]. Intra- and interobserver coefficients of variation for left atrial volume were similar, 8% and 10%, respectively. LV mass was calculated with the Devereux formula [21] and converted to *Z*-score [22]. We have previously published on associations between GDM and maternal/child adiposity and child LV mass [10], and in this manuscript, we focused on LV mass relations with LV diastolic function.

### Heart rate (HR)

Heart rate was measured at rest with electrocardiography and calculated as the mean of three heart cycles.

### Data analysis

Data are presented as mean ± SD, median (interquartile range) or as a count (percentage). All continuous variables were assessed for normal distribution based on histograms and normal Q-Q plots.

We analysed participants with reference to prenatal GDM exposure (GDM-positive vs GDM-negative). To account for the severity of GDM, we conducted further analyses based on GDM treatment (GDM-diet—treated only with diet, GDM-medicated—treated with metformin or insulin). The cohort was further divided into subgroups to analyse the separate influence of GDM, maternal pre-pregnancy obesity, and child overweight/obesity (ISO-BMI ≥ 25 kg/m^2^; age and sex-specific BMI values corresponding with adult BMI ≥ 25 kg/m^2^ [13]). Three mothers with missing information on GDM were excluded from analyses.

GDM-positive and GDM-negative groups were compared with the independent t test or Mann–Whitney U test if not normally distributed. Subgroups stratified for GDM severity (GDM-negative, GDM-diet, and GDM-medicated), or stratified for maternal pre-pregnancy obesity, child overweight/obesity, and GDM exposure were analysed with One-Way ANOVA with post hoc Tukey’s HSD, or with Kruskal–Wallis test, with post hoc pairwise comparison adjusted with Bonferroni, if not normally distributed. Two-tailed *P* value ≤ 0.05 was set as significant.

ANCOVA model with Bonferroni correction was used to assess the total effect of GDM status (GDM-negative, GDM-diet, and GDM-medicated) on child LV diastolic function with adjustment for child HR (for parameters statistically significantly associated with child HR), using two-tailed *P* value ≤ 0.05 as significant.

The associations between child LV function and participants characteristics were explored using Pearson’s correlation coefficient. Due to the risk of type I error, we applied the conservative significance threshold (P ≤ 0.01) and focused on patterns instead of single associations. Multivariable linear regression modelling was used to assess the effect of child body fat percentage on left atrial volume, adjusting for child lean body mass, age, sex, systolic BP, HR, maternal lean body mass, maternal pre-gestational BMI, and GDM status (negative = 0, positive = 1). Multicollinearity was assessed with the Variance Inflation Factor (VIF), with the maximum value of 3.

Statistical analysis was performed with SPSS, IBM, version 25.

## Results

### Participant characteristics

The summary of background characteristics including gestational glycaemia and percentage of neonatal hypoglycaemia is presented in Table 1. Maternal pre-pregnancy BMI was increased (mean ± SD; 30.7 ± 5.6 kg/m^2^), 128 women had pre-pregnancy obesity, and 96 were diagnosed with GDM in the indexed pregnancy (including 36 on metformin or insulin): 61 in first trimester, 32 in second trimester, and three in third trimester. Child BMI *Z*-score, systolic and diastolic BP *Z*-scores were elevated as compared with a healthy Finnish population (Table 1). Child body fat percentage increased by 0.3% (95% CI; 0.1, 0.5) and child BMI *Z*-score by 0.06 (95% CI 0.01, 0.11) per 1 kg/m^2^ increase in pre-pregnancy BMI, but only in the setting of maternal pre-gestational obesity (*P* = 0.02 and *P* = 0.009, respectively). Offspring BMI *Z*-score was not associated with the difference in maternal pre-pregnancy and follow-up BMI (*P* = 0.32). Offspring anthropometrics, body composition, BP, and LV mass were not related to GDM exposure, but children following GDM pregnancies had lower resting HR (Table 2). Detailed child and maternal background associations have been presented in our previous work [9, 10].

**Table 1:** Maternal and child characteristics stratified for gestational diabetes status [data are presented as mean (± SD) or as n (%)]

Variable	All	GDM-negative	GDM-positive			*P* value GDM + vs **− **
			All	Diet	Medicated	
	*N* = 201	*N* = 102	*N* = 96	*N* = 60	*N* = 36	
Mother at gestation						
Age (first trimester) [y]	33.5 ± 4.5	33.2 ± 4.7	33.9 ± 4.2	33.5 ± 4.3	34.6 ± 3.9	0.26
Pre-gestational BMI [kg/m^2^]	30.7 ± 5.6	31.1 ± 5.4	30.2 ± 5.9	29.4 ± 5.6	31.5 ± 6.3	0.26
Pre-gestational BMI ≥ 25 kg/m^2^	34 (17%)	9 (8.9%)	24 (25%)	14 (24%)	10 (28%)	**0.005**
Pre-gestational BMI ≥ 30 kg/m^2^	128 (64%)	74 (73%)	52 (55%)	31 (53%)	21 (58%)	**0.008**
First- to third-trimester weight gain [kg]	6.7 ± 4.5	7.5 ± 4.5	6.0 ± 4.5	6.4 ± 4.4	5.2 ± 4.6	**0.022**
First-trimester fasting glucose [mmol/L]	5.0 ± 0.4	4.9 ± 0.3	5.2 ± 0.4	5.1 ± 0.4	5.4 ± 0.5**	** < 0.0001**
First-trimester HbA_1C_ [% (mmol/mol)]	5.3 ± 0.3 (34 ± 3.3)	5.2 ± 0.3 (33 ± 3.3)	5.4 ± 0.3 (36 ± 3.3)	5.4 ± 0.2 (36 ± 2.2)	5.5 ± 0.2 (37 ± 2.2)	** < 0.0001**
First-trimester HOMA-IR	1.8 ± 1.2	1.7 ± 0.9	2.1 ± 1.5	1.9 ± 1.4	2.4 ± 1.6	**0.03**
Second-trimester fasting glucose [mmol/L]	4.9 ± 0.5	4.7 ± 0.3	5.1 ± 0.5	5.1 ± 0.3	**5.3** ± 0.6*	** < 0.0001**
Second-trimester HOMA-IR	2.1 ± 1.3	1.8 ± 0.8	2.4 ± 1.7	2.1 ± 1.3	2.9 ± 2.1*	**0.004**
Third-trimester fasting glucose [mmol/L]	4.8 ± 04	4.7 ± 0.3	5.0 ± 0.4	4.9 ± 0.4	5.0 ± 0.4	** < 0.0001**
Third-trimester HbA_1C_ [% (mmol/mol)]	5.5 ± 0.3 (37 ± 3.3)	5.4 ± 0.3 (36 ± 3.3)	5.6 ± 0.3 (38 ± 3.3)	5.6 ± 0.3 (38 ± 3.3)	5.6 ± 0.3 (38 ± 3.3)	**0.0001**
Third-trimester HOMA-IR	2.7 ± 1.5	2.6 ± 1.3	2.8 ± 1.6	2.6 ± 1.7	3.1 ± 1.3	0.51
Neonate						
Birth weight *Z*-score	0.22 ± 0.98	0.17 ± 1.10	0.29 ± 0.83	0.29 ± 0.82	0.29 ± 0.87	0.41
Hypoglycaemia	21 (10%)	3 (2.9%)	18 (19%)	10 (17%)	8 (22%)	**0.0003**
Child at follow-up						
Boys	111 (55%)	58 (57%)	51 (53%)	31 (52%)	20 (56%)	0.67
Age [y]	6.09 ± 0.49	6.04 ± 0.47	6.11 ± 0.48	6.21 ± 0.51	5.95 ± 0.39	0.30
Weight [kg]	23.8 ± 4.1	23.6 ± 4.3	23.9 ± 3.9	23.9 ± 3.9	23.9 ± 3.9	0.64
Height [cm]	119.1 ± 6.6	118.9 ± 6.9	119.3 ± 6.3	120.3 ± 5.9	117.7 ± 6.6	0.65
BMI *Z*-score	**0.45 ± 0.93**^**a**^	0.41 ± 1.01	0.5 ± 0.84	0.35 ± 0.77	0.76 ± 0.9	0.50
Lean body mass [kg]	16.5 ± 2.4	16.5 ± 2.5	16.6 ± 2.3	16.8 ± 2.3	16.3 ± 2.2	0.62
Fat mass [kg]	7.2 ± 2.0	7.2 ± 2.1	7.3 ± 1.9	7.1 ± 1.9	7.6 ± 2.0	0.71
Body fat [%]	30.0 ± 4.0	30.0 ± 4.1	30.2 ± 3.8	29.4 ± 3.5	31.3 ± 4.2	0.74
Systolic BP *Z*-score	**0.39 ± 0.59**^**a**^	0.35 ± 0.61	0.43 ± 0.57	0.41 ± 0.55	0.48 ± 0.60	0.31
Diastolic BP *Z*-score	**0.59 ± 0.52**^**a**^	0.58 ± 0.49	0.61 ± 0.56	0.56 ± 0.53	0.68 ± 0.60	0.74

*BMI* body mass index, *HbA*_*1C*_ glycated haemoglobin, *HOMA-IR* homeostasis model assessment of insulin resistance [fasting insulin (μU/ml) × fasting glucose (mmol/L)/22.5], *BP* blood pressure;

*P ≤ 0.05, and **P ≤ 0.001 between diet and medicated GDM (ANOVA with post hoc Tukey's HSD);

^a^*P* ≤ 0.0001 compared with the reference population

Significant results are bolded (*P* ≤ 0.05)

**Table 2:** Child heart rate, left ventricle mass, and diastolic function stratified for gestational diabetes status [data are presented as mean ± SD or median (IQR)]

Variable	All	GDM-negative	GDM-positive			*P* value GDM + vs** − **
			All	Diet	Medicated	
	*N* = 201	*N* = 102	*N* = 96	*N* = 60	*N* = 36	
Resting heart rate [bpm]	81.4 ± 8.9	83.0 ± 9.5	79.7 ± 8.0	78.5 ± 7.4	81.8 ± 8.7	**0.009**
Left ventricle mass *Z*-score	0.78 ± 0.68	0.76 ± 0.66	0.82 ± 0.70	0.82 ± 0.74	0.82 ± 0.63	0.52
Left atrial volume index (ml/m^1.48^ or ml/m^1.08^) ^a^	28.47 ± 5.72	28.5 ± 5.82	28.27 ± 5.63	28.45 ± 5.13	27.98 ± 6.44	0.78
Left atrial volume index *Z*-score	**− 0.42 ± 1.09**^**b**^	**− **0.39 ± 1.12	**− **0.48 ± 1.06	**− **0.48 ± 0.99	**− **0.48 ± 1.19	0.55
E wave [cm/s]	95.5 ± 14.6	95.4 ± 14.8	95.6 ± 14.4	93.0 ± 13.5	99.8 ± 15.1	0.93
E wave *Z*-score	**− 0.89 ± 1.05**^**b**^	**− **0.90 ± 1.07	**− **0.88 ± 1.03	**− **1.06 ± 0.97	**− **0.58 ± 1.08	0.86
A wave [cm/s]	44.3 ± 11.7	45.1 ± 12.4	43.1 ± 11.0	40.9 ± 9.2	46.7 ± 12.7	0.24
A wave *Z*-score	**− 0.76 ± 1.19**^**b**^	**− **0.7 ± 1.21	**− **0.85 ± 1.18	**− **1.02 ± 1.0	**− **0.56 ± 1.39	0.38
E/A	2.16 (0.91)	2.11 (0.80)	2.24 (1.0)	2.30 (0.83)	2.02 (1.16)	0.35
E/A *Z*-score	0.20 ± 1.16	0.14 ± 1.18	0.30 ± 1.13	0.41 ± 0.97	0.23 ± 1.33	0.35
S/D	0.81 (0.23)	0.81 (0.23)	0.82 (0.21)	0.83 (0.24)	0.81 (0.17)	0.89
Ar—A duration difference [ms]	**− **21.6 ± 26.6	**− **18.3 ± 25.8	**− **24.9 ± 27.4	**− **25.7 ± 28.2	**− **23.6 ± 26.4	0.10
A rev [m/s]	24.5 ± 6.2	25.1 ± 6.4	23.9 ± 6.1	23.0 ± 4.9	25.3 ± 7.4	0.21
Lateral E/E′	4.71 (1.40)	4.65 (1.31)	4.71 (1.61)	4.59 (1.40)	5.10 (1.78)	0.81
Lateral E/E′ *Z*-score	**− 1.05 (1.32)**^**b**^	**− **1.05 (1.30)	**− **1.05 (1.44)	**− **1.23 (1.23)	**− **0.74 (1.60)	0.91
Septal E/E′	6.68 ± 1.19	6.62 ± 1.21	6.75 ± 1.19	6.66 ± 1.10	6.89 ± 1.31	0.48
Septal E/E′ *Z*-score	**− 0.63 ± 0.93**^**b**^	**− **0.68 ± 0.96	**− **0.56 ± 0.93	**− **0.62 ± 0.86	**− **0.47 ± 1.03	0.4
Isovolumetric relaxation time TDI [ms]	45.9 ± 8.2	46.7 ± 8.2	45.2 ± 8.2	46.4 ± 8.6	43.3 ± 7.2	0.23
Circumferential early diastolic strain rate [1/s]	2.47 ± 0.47	2.48 ± 0.45	2.44 ± 0.47	2.45 ± 0.48	2.44 ± 0.45	0.56
Longitudinal early diastolic strain rate [1/s]	2.65 ± 0.39	2.67 ± 0.36	2.62 ± 0.42	2.62 ± 0.39	2.62 ± 0.46	0.40

*E/A* mitral inflow early-to-late diastolic flow ratio, *S/D* pulmonary venous systolic-to-diastolic flow ratio, *Ar* pulmonary venous atrial reversal peak velocity, *A* mitral inflow late diastolic peak velocity, *E/E′* mitral inflow early peak velocity-to-early diastolic tissue velocity ratio, *TDI* tissue Doppler imaging

^a^Body surface area <  = 1 m^2^ exponentiated to 1.48; body surface area > 1 m^2^ exponentiated 1.08(18)

^b^*P* ≤ 0.0001 compared with the reference population

Significant results are bolded (*P* ≤ 0.05)

### LV diastolic function

Offspring LV diastolic function with respect to GDM status is presented in Table 2 and Supplemental Table S2 (Online Resource 1). There were no significant differences between the groups. No differences were present after adjustment for HR in ANCOVA models or in the subanalysis stratified by sex (data not presented). E wave and A wave *Z*-scores were significantly different from the reference population, but no discrepancies were present between study groups. We observed abnormal values of conventional left ventricle diastolic function parameters (left atrial volume index *Z*-score ≥ 2, mitral inflow E/A Z-score ≤ -2, mitral septal or lateral E/E′ Z-score ≥ 2, pulmonary veins flow Ar-A duration difference ≥ 30 ms) in 19 children (9.5%), with no statistically significant differences with respect to GDM exposure (Online Resource 1)). The overlap of categories (2) was noticed only in one case.

Associations between child LV diastolic function and maternal pre-pregnancy BMI and gestational glycaemia were weak and incoherent, without any trends (Online Resource 1, Supplemental Table S4). The gestational stage at onset of GDM (first vs second trimester), the magnitude of maternal pre-pregnancy adiposity (normal vs overweight vs obese) or adjustment of pre-pregnancy BMI for first- to third-trimester weight gain displayed no effect on child LV diastolic function (data not shown).

We further analysed the separate influence of maternal pre-pregnancy obesity, GDM exposure, and child overweight/obesity on child LV diastolic function (Online Resource 1). In these analyses, we observed a trend toward higher values of child left atrial volume index *Z*-score in children with ISO-BMI ≥ 25 kg/m^2^ but it did not reach statistical significance.

Longitudinal early diastolic strain rate was inversely correlated with child lean body mass and BMI *Z*-score (*r* = -0.306, *P* < 0.0001 and *r* = -0.239, *P* = 0.002, respectively). Pulmonary venous S/D ratio and A′lat *Z*-score weakly correlated with child body fat percentage (*r* = 0.188; *P* = 0.008 and *r* = 0.209; *P* = 0.007, respectively, Online Resource 1). Both parameters were concurrently associated with left atrial volume index and its *Z*-score.

Child LV mass *Z*-score correlated with septal E/E′ *Z*-score and inversely with septal E′ *Z*-score (*r* = 0.246, *P* = 0.001 and *r* = -0.235, *P* = 0.001, respectively, Online Resource 1).

Child left atrial size correlated with child and maternal body size and composition, which displayed significant multicollinearity (Online Resource 1, Supplemental Fig. S2a and Fig. S2b, Supplemental Table S1). After adjusting for child lean body mass, age, sex, systolic BP, resting HR, and maternal lean body mass, pre-gestational BMI, and GDM status, child left atrial volume increased by 0.3 (0.1, 0.5) ml for each 1% of child body fat (Table 3). Child sex did not moderate the effect of lean body mass or body fat percentage on left atrial volume.

**Table 3:** Multivariable linear regression model of child left atrial volume (adjusted R2 = 0.331; *P* value < 0.001)

Variables	Beta (95% CI)	Standardized beta	*P* value
Constant	26.196 (9.574, 42.819)	–	**0.002**
Child body fat [%]	0.298 (0.079, 0.517)	0.218	**0.008**
Child lean body mass [kg]	1.233 (0.779, 1.686)	0.550	** < 0.001**
Child systolic BP [mmHg]	**− **0.139 (**− **0.241, **− **0.038)	**− **0.178	**0.007**
Child heart rate [1/minute]	**− **0.092 (**− **0.164, **− **0.020)	**− **0.167	**0.012**
Child age [*y*]	**− **1.368 (**− **3.222, 0.486)	**− **0.123	0.15
Child sex [Boy = 0, Girl = 1]	**− **0.886 (**− **2.640, 0.867)	**− **0.080	0.32
Maternal pre-pregnancy BMI [kg/m^2^]	**− **0.064 (**− **0.207, 0.079)	**− **0.066	0.377
Maternal lean body mass [kg]	0.004 (**− **0.127, 0.135)	0.005	0.957
Gestational diabetes [No = 0, Yes = 1]	**− **0.180 (**− **1.492, 1.132)	**− **0.016	0.787

*CI* Confidence Interval, *BMI* body mass index

Significant results are bolded (*P* ≤ 0.05)

### LV systolic function

Analysis regarding LV systolic function and GDM status is presented in Table 4. Child LV basal circumferential systolic function assessed with strain echocardiography was minimally decreased in children following GDM (peak strain *Z*-score, *P* = 0.016), with no further decrease in more severe GDM forms. No differences were observed with respect to circumferential function assessed with conventional echocardiography or longitudinal systolic function. Maternal pre-pregnancy BMI and gestational glucose levels were not associated with child LV systolic function (Online Resource 1). The gestational stage at onset of GDM (first vs second trimester), the magnitude of maternal pre-pregnancy adiposity (normal vs overweight vs obese), or adjustment of pre-pregnancy BMI for first- to third-trimester weight gain displayed no effect on child LV systolic function (data not shown). We found no associations between LV systolic function and child adiposity or glycaemia (Online Resource 1).

**Table 4:** Child left ventricle systolic function stratified for gestational diabetes status [data are presented as mean ± SD or as median (IQR)]

Variable	All	GDM-negative	GDM-positive			*P* value GDM + vs −
			All	Diet	Medicated	
	*N* = 201	*N* = 102	*N* = 96	*N* = 60	*N* = 36	
Fractional shortening [%]	35.7 ± 3.2	35.8 ± 3.2	35.4 ± 3.2	34.9 ± 3.2	36.3 ± 2.9	0.40
Ejection fraction [%]	63.6 ± 3.8	63.6 ± 3.4	63.5 ± 4.0	62.9 ± 4.0	64.4 ± 4.0	0.90
Lateral S′ [cm/s]	10.2 ± 1.9	10.2 ± 1.8	10.3 ± 1.9	10.2 ± 1.8	10.4 ± 1.8	0.66
Lateral S′ *Z*-score	0.20 ± 1.04	0.17 ± 1.01	0.22 ± 1.09	0.29 ± 1.01	0.11 ± 1.22	0.77
Septal S′ [cm/s]	7.7 ± 0.8	7.8 ± 0.8	7.7 ± 0.9	7.7 ± 1.0	7.6 ± 0.7	0.40
Septal S′ *Z*-score	**− **0.53 (1.25)	**− **0.53 (1.25)	**− **0.53 (1.21)	**− **0.52 (1.22)	**− **1.0 (1.23)	0.36
Longitudinal peak systolic strain [%]	**− **21.7 ± 2.1	**− **21.9 ± 2.0	**− **21.4 ± 2.2	**− **21.1 ± 2.2	**− **21.9 ± 2.2	0.13
Longitudinal peak systolic strain *Z*-score	0.72 ± 1.24^a^	0.85 ± 1.19	0.57 ± 1.28	0.41 ± 1.27	0.85 ± 1.26	0.13
Longitudinal systolic strain rate [1/s]	**− **1.32 ± 0.21	**− **1.33 ± 0.15	**− **1.32 ± 0.15	**− **1.30 ± 0.16	**− **1.35 ± 0.14	0.73
Basal circumferential peak systolic strain [%]	**− **22.5 ± 2.2	**− **22.8 ± 2.2	**− **22.1 ± 2.2	**− **22.0 ± 2.2	**− **22.2 ± 1.9	**0.016**
Basal circumferential peak systolic strain *Z*-score	1.35 ± 1.23^a^	1.53 ± 1.23	1.11 ± 1.16	1.07 ± 1.23	1.16 ± 1.07	**0.015**
Basal circumferential systolic strain rate [1/s]	**− **1.42 ± 0.16	**− **1.44 ± 0.17	**− **1.39 ± 0.14	**− **1.37 ± 0.13	**− **1.41 ± 0.15	**0.010**

^a^*P* ≤ 0.0001 compared with the reference population

Significant results are bolded (*P* ≤ 0.05)

No significant associations were found in the analysis of maternal obesity, GDM exposure, and child overweight/obesity on child LV systolic function (Online Resource 1).

## Discussion

This study provides new insights into the field of prenatal conditioning of offspring cardiovascular health. We observed no effect of gestational hyperglycaemia or GDM on child LV diastolic or systolic function at 6 years of age. Our results extend current knowledge, as the longest prospective follow-up reported so far is limited to a relatively small cohort of 3-year-old children (29 following GDM) with no data available on LV systolic function [23].

Child left atrial volume is strongly related to body size, but can enlarge in the setting of LV diastolic dysfunction. The present study results are consistent with this but show a small independent association between left atrial volume and adiposity. The increase in left atrial volume attributed to child adiposity was clinically silent, and its long-term consequences for left ventricle diastolic function remain at present unknown. However, our findings may indicate a need for longitudinal evaluation due to possible progression. We also demonstrated associations between child adiposity and A′lat and pulmonary venous S/D ratio, which we speculate to be secondary to the increased left atrial volume. Our study extends the knowledge of early cardiac adaptation in the setting of mild overweight in children, as previous research focused largely on clinical obesity [24, 25].

Our study shows an association between child adiposity and maternal pre-pregnancy obesity. Taking into consideration the associations between child adiposity and left atrial volume, the prevention of early weight gain in the offspring of mothers with obesity should be further evaluated as a strategy to reduce long-term cardiovascular risks. The effect of maternal pregravida obesity on child body composition could be attributed to common diet behaviours, but we observed no association between offspring adiposity and maternal BMI change from pre-pregnancy to follow-up. We conclude that maternal pregravida obesity, but not GDM could play a role in the transgenerational conditioning of offspring cardiovascular health.

We observed a negative correlation between child LV mass *Z*-score and septal but not lateral myocardial relaxation in the normally structured hearts of the present study. Although no overt septal hypertrophy was found, we hypothesize that milder septal thickening could potentially negatively influence myocardial relaxation.

Despite normal LV diastolic function in the majority of participants (based on clinical judgement), we report significantly lower mitral inflow peak velocities (E and A-wave *Z*-scores), but no differences between study groups. E/A ratio *Z*-score was close to the population mean. Similar results were previously reported with the same normalization method in a population of children at three years of age [23]. Taking into consideration that *Z*-score references were not validated in an external population, the observed discrepancies could reflect the selection bias in the original study [18]. Similarly, decreased values of left atrial volume index *Z*-score were consistent between study subgroups. This could be explained as the effect of adiposity related over-indexing, because children with body size exceeding the 95th percentile were excluded from the reference population [20].

The echocardiographic assessment of LV diastolic function in children is challenging due to the lack of a standardized and validated approach (available recommendations have not uniformly covered and agreed upon diastolic dysfunction grading in the paediatric population [26, 27]), but echocardiographic assessments integrating Doppler, Tissue Doppler, and left atrial volume are commonly used in the clinical setting [28, 29]. Thus, we decided to abandon any subjective grading and by nature controversial definition of child LV diastolic dysfunction and include a more comprehensive analysis with echocardiographic variables commonly used in the clinical and research practice.

We observed minimally reduced systolic circumferential LV deformation characteristics in children following GDM (no further decline was noted in more severe GDM forms), but gestational glycaemia, maternal pre-pregnancy obesity, and child adiposity were not associated with LV systolic function (including speckle tracking and conventional parameters). These observed associations should be interpreted cautiously as type I error cannot be excluded.

The cardiovascular development has a multifactorial background, including genetic factors, the intrauterine milieu, and the postnatal environment. The precise distinction between these factors is challenging, but highly homogenous study groups, with except for GDM, allowed us to conclude on foetal programming related to GDM exposure. The differences in gestational glycaemia between study subgroups were mild and could presumably be attributed to the treatment, but foetal steal phenomenon could play a role [30]. This increases the generalizability of our findings, as mild, well-controlled GDM is the most prevalent form in developed countries. However, our results may not apply to more severe forms of GDM or pre-gestational diabetes. The lack of a formal control group (women without increased risk of GDM) is the shortcoming, but the present cohort nevertheless included a subgroup of women without obesity and GDM in the indexed pregnancy (*N* = 27), which should be considered as normal references with regard to exposure. The study sample size of 200 dyads was assumed to be large enough to find clinically relevant differences between GDM groups and allow the longitudinal assessment of the cohort beyond 6 years. A priori defining of significant differences with regard to the comprehensive assessment of LV function was challenging, but the post hoc analysis provided a power of 0.8 to detect a difference of 0.5 SD between groups at an *α*-level of 0.01. Importantly, our results are confined to early childhood and further growth, including additional weight gain or the appearance of other cardiometabolic risk factors, could influence the findings later on. We were unable to control for lifestyle factors (e.g. diet, physical activity) and fathers’ characteristics. Another limitation is the lack of cardiovascular data from the neonatal period, which precludes longitudinal analysis at this point, but we aim to reevaluate this cohort later on.

## Conclusions

No plausible evidence of foetal cardiovascular programming on cardiac function attributed to GDM was found in early childhood. Although child left atrial volume was determined mainly by child lean body mass, we found a small independent association with adiposity as well in our offspring population with early weight gain. Maternal pre-gestational obesity seems to be reflected in the child’s body composition, implicitly increasing long-term cardiovascular risks.

## Electronic supplementary material

Below is the link to the electronic supplementary material.

Supplementary file1 (PDF 583 kb)
