# Supplementary material for: No effect of gestational diabetes or pre-gestational obesity on 6-year offspring left ventricular function—RADIEL study follow-up
Source: Acta Diabetol. 2020 Jul 28;57(12):1463–72. doi: 10.1007/s00592-020-01571-z (PMC7591425; doi:10.1007/s00592-020-01571-z)
Supplement: Supplementary file 1 — Supplementary file1 (PDF 583 kb) [file 592_2020_1571_MOESM1_ESM.pdf]

Supplemental Material

Re: No effect of gestational diabetes or pre-gestational obesity on 6-year offspring left ventricular function—RADIEL study follow-up

Linda Litwin[a,b] MD (linda.litwin@med.sum.edu.pl)
Johnny K.M. Sundholm[a] MD, PhD (johnny.sundholm@helsinki.fi)
Kristiina Rönö[c] MD, PhD (kristiina.rono@helsinki.fi)
Saila B. Koivusalo[c] MD, PhD (saila.koivusalo@helsinki.fi)
Johan G. Eriksson[d,e,f] MD, Professor (johan.eriksson@helsinki.fi)
Taisto Sarkola[a,g] MD, PhD (taisto.sarkola@helsinki.fi)

[a] Children's Hospital, University of Helsinki and Helsinki University Hospital, Helsinki, Finland
[b] Department of Congenital Heart Defects and Pediatric Cardiology, FMS in Zabrze, Medical University of Silesia, Katowice, Poland
[c] Women's Hospital, University of Helsinki and Helsinki University Hospital, Helsinki, Finland
[d] University of Helsinki and Helsinki University Hospital, Helsinki, Finland
[e] Folkhälsan Research Center, Helsinki, Finland
[f] Yong Loo Lin School of Medicine, National University of Singapore, Singapore
[g] Minerva Foundation Institute for Medical Research, Helsinki, Finland

Address for Correspondence:

Linda Litwin, MD, linda.litwin@med.sum.edu.pl,

University of Helsinki, P.O.Box 281, FIN-00029 HUS, Helsinki, Finland.

Permanent address: Department of Congenital Heart Defects and Pediatric Cardiology, Medical University of Silesia, M.Sklodowskiej-Curie 9, 41-800 Zabrze, Poland,

Phone: +48 322713401; Fax: +48 322713401

Content:

A. **Methods details.**
B. **Supplemental Tables.**
C. **Supplemental Figures.**
D. **References.**

A. Methods details

Body composition:

Excellent concordance has been documented between bioimpedance and dual-energy X-ray absorptiometry in adults, but a systematic underestimation of body fat percentage has been reported in children [1, 2]. Child lean body mass was measured by bioelectrical impedance (InBody 720, InBody Bldg, Korea) and calculated with the previously validated formula (based on age, sex, height, weight, and BMI Z-score) [3]. Lean body mass measurements derived with both methods were strongly correlated (r=0.951). However, the body fat percentage assessed by bioimpedance method was lower (mean difference ± SD; -13.6% ± 3.8%) and the difference was inversely correlated with child weight (r=-0.38; p<0.0001).

Echocardiography

Images were stored in the raw DICOM-format and analysed offline with EchoPAC GE Healthcare (version 113). The assessment of LV diastolic and systolic function included: left atrial volume with the biplane area-length method (indexed to body surface area and converted to Z-scores in reference to recent paediatric data [4]), mitral inflow early-to-late diastolic flow (E/A) ratio, pulmonary venous systolic-to-diastolic peak velocity ratio (S/D), pulmonary venous A wave reversal (Ar) amplitude and duration, time difference between Ar and mitral A duration, isovolumic relaxation time with Tissue Doppler, mitral lateral and septal peak early and late diastolic tissue velocities (E′lat, E′med, A'lat, A'med), mitral E-to-E'med and E-to E-'lat, mitral lateral and septal systolic tissue velocities (S'lat, S'med), fractional shortening from M-mode recordings, ejection fraction with the Simpson's biplane method, mitral annular plane systolic excursion (MAPSE), strain (systolic) and strain rates (systolic and early diastolic) with 2D speckle tracking (circumferential from the parasternal short axis view at the level of mitral valve leaflets and longitudinal from apical 4-chamber and 2-chamber views) according to guidelines. The results were normalized for body surface area and converted to Z-scores in reference to recent high-quality paediatric data, obtained with the same echocardiography protocol, matched for child race and age [5, 6].

B. Supplemental Tables:

a. Supplemental Table S1. Correlation analyses for determinants of child left atrial volume at six years postpartum (Pearson correlation coefficient).
b. Supplemental Table S2. Child left ventricle diastolic function stratified for gestational diabetes status [data are presented as mean ± SD or median (IQR)].
c. Supplemental Table S3. Left ventricle diastolic function abnormal parameters stratified for gestational diabetes status [data are presented as count].
d. Supplemental Table S4. Associations of child left ventricle diastolic function parameters at six years of age with maternal pre-pregnancy adiposity and gestational glycemia (Pearson correlation coefficient).
e. Supplemental Table S5. Child left ventricle diastolic function parameters associations with body composition, blood pressure, resting heart rate, left ventricle mass, and glucose levels at six years of age (Pearson correlation coefficient).
f. Supplemental Table S6. Child diastolic function at six years of age comparison between groups stratified for maternal pre-pregnancy obesity and child overweight or obesity (mean ± SD or count (%)).
g. Supplemental Table S7. Child diastolic function at six years of age comparison between groups stratified for maternal GDM and pre-pregnancy obesity (mean ± SD or count (%)).
h. Supplemental Table S8. Child diastolic function at six years of age comparison between groups stratified for maternal GDM and child overweight or obesity (mean ± SD or count (%)).
i. Supplemental Table S9. Associations of child left ventricle systolic function at six years of age with maternal pre-pregnancy adiposity and gestational glycemia (Pearson correlation coefficient).
j. Supplemental Table S10. Child left ventricle systolic function associations with body composition, blood pressure, resting heart rate, left ventricle mass, and glucose levels at six years of age (Pearson correlation coefficient).
k. Supplemental Table S11. Child left ventricle systolic function comparison between groups stratified for maternal pre-pregnancy obesity and child overweight or obesity (data are presented as means ± SD or median (IQR)).
l. Supplemental Table S12. Child left ventricle systolic function comparison between groups stratified for maternal GDM and pre-pregnancy obesity (data are presented as means ± SD or median (IQR)).
m. Supplemental Table S13. Child left ventricle systolic function comparison between groups stratified for maternal GDM and child overweight or obesity (mean ± SD).

Supplemental Table S1. Correlation analyses for determinants of child left atrial volume at six years postpartum (Pearson correlation coefficient).

Variable	r	p-value
Child		
**Age**	0.158	0.03
**Height**	0.423	**< 0.0001**
**Weight**	0.529	**< 0.0001**
**BMI**	0.422	**< 0.0001**
**BMI Z-score**	0.426	**< 0.0001**
**Waist-hip ratio**	-0.018	0.81
**Waist-height ratio**	0.162	0.02
**Lean body mass**	0.511	**< 0.0001**
**Fat mass**	0.458	**< 0.0001**
**Body fat percentage**	0.248	**0.001**
**Birth weight**	0.219	**0.002**
**Birth crown-heel length**	0.228	**0.001**
**Systolic blood pressure**	0.022	0.762
**Systolic blood pressure Z-score**	-0.143	0.04
**Diastolic blood pressure**	-0.016	0.82
**Diastolic blood pressure Z-score**	-0.145	0.04
**Fasting glucose**	0.032	0.68
**HbA_{1c}**	-0.073	0.35
**Insulin**	0.075	0.35
**Total cholesterol**	-0.31	0.7
**LDL cholesterol**	-0.28	0.72
**HDL cholesterol**	-0.34	0.67
**Total triglycerides**	-0.011	0.89
Mother		
**Height**	0.198	**0.005**
**Weight**	0.127	0.08
**BMI**	0.058	0.42
**Waist-hip ratio**	0.066	0.36
**Lean body mass**	0.275	**< 0.0001**
**Fat mass**	0.097	0.18
**Body fat percentage**	-0.005	0.94
**Pre-gestational BMI**	0.075	0.29
**Systolic blood pressure**	-0.006	0.94
**Diastolic blood pressure**	-0.012	0.87
**Fasting glucose 1st trimester**	0.065	0.37
**HbA_{1c} 1st trimester**	0.064	0.41
**HOMA-IR 1st trimester**	0.141	0.06
**Fasting glucose 2nd trimester**	-0.003	0.96
**HOMA-IR 2nd trimester**	0.044	0.55
**Fasting glucose 3rd trimester**	-0.11	0.14
**HbA_{1c} 3rd trimester**	-0.103	0.18
**HOMA-IR 3rd trimester**	0.014	0.85

BMI – body mass index; **HbA_{1C}** – glycated haemoglobin; **HOMA-IR** – homeostasis model assessment of insulin resistance [fasting insulin (μU/ml) × fasting glucose (mmol/L)/22.5]; Significance of the data in bold $P < 0.01$

Supplemental Table S2. Child left ventricle diastolic function stratified for gestational diabetes status [data are presented as mean ± SD or median (IQR)].

Variable	All	GDM negative	GDM positive			P-value GDM + vs -
			All	Diet	Medicated	
	N=201	N=102	N=96	N=60	N=36	
Left atrial volume [ml]	23.65 ± 5.47	23.56 ± 5.7	23.63 ± 5.27	23.94 ± 5.02	23.13 ± 5.71	0.93
S wave [cm/s]	49.6 ± 8.6	49.9 ± 8.7	49.3 ± 8.6	49.2 ± 9.6	49.5 ± 7.8	0.62
S wave Z-score	**0.22 ± 0.73 §**	0.24 ± 0.74	0.19 ± 0.74	0.15 ± 0.73	0.2 ± 0.53	0.64
D wave [cm/s]	60.6 ± 8.6	60.8 ± 9.3	60.3 ± 7.9	59.2 ± 7.7	62.1 ± 8.0	0.68
D wave Z-score	-0.02 ± 0.79	0.0 ± 0.85	-0.05 ± 0.73	-0.15 ± 0.71	0.13 ± 0.73	0.66
Lateral E' [cm/s]	19.7 ± 2.5	19.6 ± 2.6	19.8 ± 2.4	20.1 ± 2.4	19.4 ± 2.4	0.49
Lateral E' Z-score	0.19 ± 0.95	0.16 ± 0.97	0.23 ± 0.94	0.33 ± 0.94	0.08 ± 0.93	0.59
Lateral A' [cm/s]	6.42± 1.3	6.5 ± 1.2	6.4 ± 1.4	6.2 ± 1.3	6.7 ± 1.4	0.67
Lateral A' Z-score	-0.05 (0.73)	-0.05 (0.72)	-0.05 (1.51)	-0.06 (1.54)	0.64 (0.72)	0.69
Septal E' [cm/s]	14.5 ± 1.7	14.6 ± 1.7	14.3 ± 1.7	14.0 ± 1.7	14.7 ± 1.7	0.14
Septal E' Z-score	**-0.33 ± 0.8 §**	-0.25 ± 0.78	-0.43 ± 0.81	-0.57 ± 0.78	-0.2 ± 0.82	0.12
Septal A' [cm/s]	6.0 (1.0)	6.0 (1.0)	6.0 (1.0)	6.0 (1.0)	6.0 (2.0)	0.98
Septal A' Z-score	**-0.05 (1.01) §**	-0.06 (1.01)	-0.05 (1.02)	-0.06 (1.02)	-0.05 (1.82)	0.82
Isovolumetric relaxation time CWD [ms]	56.7 ± 12.7	56.9 ± 12.8	56.6 ± 12.8	58.3 ± 13.6	53.7 ± 10.8	0.89

CWD – continuous wave Doppler; § P ≤0.0001 compared with the general population; Significant results are bolded (P ≤ 0.05)

Supplemental Table S3. Left ventricle diastolic function abnormal parameters stratified for gestational diabetes status [data are presented as count].

Variable	All	GDM negative	GDM positive			* P-value GDM + vs -
			All	Diet	Medicated	
	N=201	N=102	N=96	N=60	N=36	
**Left atrial volume index Z-score ≥ 2**	5	5	0	0	0	0.06
**E/A Z-score ≤ -2**	4	3	1	1	0	0.62
**E/E'med Z-score ≥ 2**	2	1	1	1	0	1.0
**E/E'lat Z-score ≥ 2**	3	3	0	0	0	0.25
**Ar-A ≥ 30 [ms]**	5	3	2	1	1	1.0

E/A – mitral inflow early-to-late peak velocity ratio; **E/E'med** – mitral inflow early diastolic peak velocity to mitral early diastolic tissue peak velocity; **E/E'lat** – mitral inflow early diastolic peak velocity to mitral early diastolic tissue peak velocity; ***** – Fisher's Exact Test (two-sided P-value).

Supplemental Table S4. Associations of child left ventricle diastolic function parameters at six years of age with maternal pre-pregnancy adiposity and gestational glycemia (Pearson correlation coefficient).

	Pre-pregnancy BMI	1st trimester HbA_{1C}	3rd trimester HbA_{1C}	1st trimester HOMA-IR	2nd trimester HOMA-IR	3rd trimester HOMA-IR
Left atrial volume [ml]	.075	.064	-.103	.141	.044	.014
Left atrial volume index ($ml/m^{1.48}$ or $ml/m^{1.08}$) ||	-.072	-0.9	**-.205** †	.04	-.017	-.038
Left atrial volume index Z-score	-.003	-,033	-.154	0,089	.03	.001
E wave [cm/s]	-.011	.04	-.002	.01	.027	-.024
E wave z-score	.011	.061	.005	.028	.039	-.014
A wave [cm/s]	.015	-.045	-.194	-.015	-.014	-.083
A wave Z-score	.041	-.018	-.189	.031	.006	-.056
E/A	.010	.076	.187	-.018	.021	.036
E/A Z-score	-.029	.054	.175	-.014	.013	.038
S wave [cm/s]	.042	.032	.013	.063	.048	-.034
S wave Z-score	.054	.058	.027	.078	.06	-.046
D wave [cm/s]	.078	.172	.154	.081	.147	.134
D wave Z-score	.072	.166	.151	.076	.143	.132
S/D	-.006	-.091	-.079	-.004	-.057	-.104
Lateral E' [cm/s]	-.015	.07	.092	-.034	-.026	.01
Lateral E' Z-score	-.033	.042	.084	-.053	-.042	.006
Lateral A' [cm/s]	.09	-.006	-.08	.125	-.011	.022
Lateral A' Z-score	.075	-.001	-.078	.125	-.032	.004
Lateral E/E'	.013	-.031	-.127	.041	-.076	-.025
Lateral E/E' Z-score	.007	-.009	-.077	.04	.044	-.035
Septal E' [cm/s]	.121	.083	.126	.036	**.218** †	.135
Septal E' Z-score	.098	.053	.117	.017	**.201** †	.127
Septal A' [cm/s]	.021	-.022	-.191	.093	-.022	.041
Septal A' Z-score	.02	-.027	**-.2** †	.107	-.029	.042
Septal E/E'	-.097	-.021	-.082	-.024	-.107	-.111
Septal E/E' Z-score	-.076	.015	-.068	-.001	-.095	-.11
Isovolumetric relaxation time CWD [ms]	.102	-.141	-.135	.027	-.048	.036
Isovolumetric relaxation time TDI [ms]	-.034	**-.205**†	-.172	-.074	-.056	-.001
Circumferential early diastolic strain rate [1/s]	-.038	.039	.052	-.042	-.041	-.031
Longitudinal early diastolic strain rate [1/s]	-.089	-.083	-.201	-.057	-.012	-.030

BMI – body mass index; **HbA_{1C}** – glycated haemoglobin; **HOMA-IR** – homeostasis model assessment of insulin resistance [fasting insulin (μU/ml) × fasting glucose (mmol/L)/22.5]; † **p<0.01, and ‡ p<0.001 (two-tailed);** || – BSA <=1 m^2 exponentiated to 1.48; BSA > 1 m^2 exponentiated 1.08[4]; Significance of the data in bold P < 0.01

Supplemental Table S5. Child left ventricle diastolic function parameters associations with body composition, blood pressure, resting heart rate, left ventricle mass, and glucose levels at six years of age (Pearson correlation coefficient).

	BMI z-score	Body fat %	Lean body mass	SBP z-score	DBP z-score	Heart rate	LVM z-score	HbA_{1C}	Plasma glucose	Insulin
Left atrial volume [ml]	**.426 ‡**	**.248 ‡**	**.511 ‡**	-.143	-.145	**-.269 ‡**	.107	-.073	.032	.075
Left atrial volume index ($ml/m^{1.48}$ or $ml/m^{1.08}$) ‖	-.017	-0.47	-.149	**-.215 †**	-.169	-.156	.101	-.209 †	-.05	-.08
Left atrial volume index Z-score	.130	.087	.053	-.181	-.134	**-.190** **	.087	-.150	-.044	-.047
E wave [cm/s]	.026	.055	-.096	**.292 ‡**	.122	-.083	.096	.054	.067	-.07
E wave z-score	.102	.105	.015	**.296 ‡**	.122	-.109	.100	.070	.081	-.047
A wave [cm/s]	.055	.068	-.125	.143	**.247 ‡**	**.476 ‡**	-.145	-.102	.016	.037
A wave Z-score	.170	.156	.048	.133	**.241** **	**.428 ‡**	-.126	-.096	.04	.085
E/A	-.023	-.047	.069	.038	-.148	**-.436 ‡**	.159	.164	.016	-.061
E/A Z-score	-.089	-.084	-.014	.022	-.172	**-.458 ‡**	.173	.120	.001	-.101
S wave [cm/s]	.064	.131	-.06	-.023	-.02	.092	.057	.11	**.244 †**	.183
S wave Z-score	.115	.158	.014	-.005	-.007	0.079	.065	.123	**.231 †**	.190
D wave [cm/s]	-.143	-.129	-.038	.135	-.029	-.153	-.013	.095	.016	.023
D wave Z-score	-.162	-.142	.009	.133	-.029	-.147	-.014	.091	.013	.017
S/D	.154	**.209 †**	-.081	-.092	.028	**.191 †**	.06	.041	**.209 †**	.136
Lateral E’ [cm/s]	.001	-0.14	.122	-.12	-.127	-.107	-.114	-.045	.014	-.026
Lateral E’ Z-score	-.09	-.064	-.007	-.123	-.118	-.083	-.118	-.063	-.001	-.058
Lateral A’ [cm/s]	**.22 †**	**.191 †**	.043	-.11	-.029	**.233 †**	-.008	.099	-.034	-.008
Lateral A’ Z-score	**.222 †**	**.188 †**	.045	-.089	-.019	**.229 †**	-.007	-.091	-.013	.004
Lateral E/E’	.005	.015	-.143	**.274 ‡**	**.207 †**	.005	.169	.04	.048	-.029
Lateral E/E’ Z-score	.094	.084	-.035	**.296 ‡**	.166	-.026	.146	.079	.041	-.01
Septal E’ [cm/s]	.03	.036	.029	-.039	-.154	.053	**-.233 †**	-.099	.043	-.038
Septal E’ Z-score	-.06	-.015	-.102	-.046	-.147	.079	**-.235 †**	-.115	.029	-.067
Septal A’ [cm/s]	.102	.051	-.04	-.02	.01	**.367 ‡**	-.011	.079	.02	.091
Septal A’ Z-score	.121	.061	-.011	-.009	.008	**.367 ‡**	.002	.09	.026	.102
Septal E/E’	-.002	.022	-.106	**.245 ‡**	.180	-.098	**.242 ‡**	.108	.028	-.031
Septal E/E’ Z-score	.091	.078	.037	**.265 ‡**	.181	-.127	**.246 ‡**	.132	.046	-.002
Isovolumetric relaxation time CWD [ms]	.09	.10	.171	.009	.082	-.018	.076	-.072	.007	.041
Isovolumetric relaxation time TDI [ms]	-.150	-.066	-.174	-.097	.056	-.109	0.008	-.063	.031	.015
Circumferential early diastolic strain rate [1/s]	-.065	-.006	-.183	-.011	-.111	.017	-.019	-.061	-.170	-.102
Longitudinal early diastolic strain rate [1/s]	**-.239 †**	-.169	**-.306 ‡**	.005	-.039	.086	-.045	-.098	-.196	-.227

BMI – body mass index; **LVM** – left ventricle mass; **SBP** – systolic blood pressure; **DBP** – diastolic blood pressure; **HbA_{1C}** – glycated haemoglobin; **SR** – strain ratio; **† p<0.01, and ‡ p<0.001** (two-tailed); ‖ BSA <=1 m^2 exponentiated to 1.48; BSA > 1 m^2 exponentiated 1.08[4]; Significance of the data in bold P < 0.01

Supplemental Table S6. Child diastolic function at six years of age comparison between groups stratified for maternal pre-pregnancy obesity and child overweight or obesity (mean ± SD or count (%)).

	Iso-BMI < 25 / BMI < 30<br>A	iso-BMI < 25 / BMI ≥ 30<br>B	iso-BMI ≥ 25 / BMI <30<br>C	iso-BMI ≥ 25 / BMI ≥ 30<br>D	ANOVA<br>P-value	D vs A P-value	D vs B P-value
N	55	89	16	39			
Left atrial volume index (ml/m$^{1.48}$ or ml/m$^{1.08}$) ‖	28.8 ± 5.6	28.4 ± 6.2	28.8 ± 4.6	28.1 ± 5.2	0.945		
Left atrial volume index z-score	-0.48 ± 1.0	-0.52 ± 1.12	-0.28 ± 0.95	-0.12 ± 1.18	0.24		
E wave Z-score	-0.88 ± 1.05	-1.02 ± 1.06	-0.95 ± 0.71	-0.6 ± 1.12	0.225		
A wave Z-score	-0.86 ± 1.13	-0.81 ± 1.21	-0.64 ± 1.32	-0.46 ± 1.13	0.368		
E/A Z-score	0.3 ± 1.06	0.17 ± 1.18	0.11 ± 1.13	0.12 ± 1.25	0.868		
S wave Z-score	0.24 ± 0.72	0.13 ± 0.75	0.35 ± 0.64	0.32 ± 0.75	0.458		
D wave Z-score	-0.09 ± 0.77	0.13 ± 0.8	-0.18 ± 0.79	-0.16 ± 0.78	0.157		
S/D ratio	0.85 ± 0.14	0.8 ± 0.19	0.87 ± 0.16	0.86 ± 0.19	0.224		
Lateral E' Z-score	0.22 ± 0.99	0.18 ± 0.81	0.32 ± 1.32	0.13 ± 1.04	0.917		
Lateral A' Z-score	0.07 ± 0.82	-0.02 ± 0.99	0.31 ± 0.68	0.64 ± 0.83	**0.002**	**0.017**	**0.001**
Lateral E/E' Z-score	-0.87 ± 1.04	-0.96 ± 0.96	-1.02 ± 0.89	-0.68 ± 1.11	0.534		
Septal E' Z-score	-0.33 ± 0.8	-0.24 ± 0.73	-0.67 ± 1.0	-.3 ± 0.73	0.254		
Septal A' Z-score	-0.38 ± 1.0	-0.51 ± 0.98	-0.35 ± 0.98	-0.15 ± 0.9	0.315		
Septal E/E' Z-score	-0.59 ± 0.96	-0.78 ± 0.92	-0.4 ± 0.69	-0.45 ± 1.01	0.209		
Abnormal inflow pattern	-	3 (3.4)	-	1 (2.6)	-		
Pseudo-normal inflow pattern	1 (1.8)	1 (1.1)	-	2 (5.1)	-		
Left atrial enlargement	-	2 (2.2)	1 (6.3)	2 (5.1)	-		
Diastolic dysfunction signs	1 (1.8)	6 (6.7)	1 (6.3)	5 (12.8)	-		

The cohort was divided into four groups based on child overweight or obesity (iso-BMI ≥ 25 kg/m^2) and maternal obesity (pre-pregnancy BMI ≥ 30 kg/m^2); values are presented as counts or means (± SD); p-value was calculated with one-way ANOVA with post-hoc Tukey HSD. **BMI** – body mass index; Significance of the data in bold P < 0.01

Supplemental Table S7. Child diastolic function at six years of age comparison between groups stratified for maternal GDM and pre-pregnancy obesity (mean ± SD or count (%)).

	**GDM- / BMI < 30**	**GDM- / BMI ≥ 30**	**GDM+ / BMI < 30**	**GDM+ / BMI ≥ 30**	**ANOVA P-value**
N	27	74	43	52	
Left atrial volume index (ml/m$^{1.48}$ or ml/m$^{1.08}$) ||	29.2 ± 5.5	28.2 ± 6.0	28.5 ± 5.4	28.2 ± 5.8	0.89
Left atrial volume index z-score	-0.34 ± 1.07	-0.41 ± 1.15	-0.5 ± 0.96	-0.43 ± 1.15	0.941
E wave z-score	-1.01 ± 1.04	-0.86 ± 1.09	-0.83 ± 0.95	-0.94 ± 1.1	0.882
A wave z-score	-0.63 ± 1.17	-0.69 ± 1.2	-0.95 ± 1.17	-0.76 ± 1.2	0.64
E/A z-score	0.02 ± 0.99	0.16 ± 1.23	0.42 ± 1.1	0.18 ± 1.17	0.502
S wave z-score	0.21 ± 0.72	0.24 ± 0.75	0.3 ± 0.7	0.11 ± 0.77	0.625
D wave z-score	-0.18 ± 0.81	0.07 ± 0.87	-0.06 ± 0.77	-0.02 ± 0.7	0.554
S/D ratio	0.86 ± 0.15	0.83 ± 0.21	0.85 ± 0.14	0.81 ± 0.17	0.597
Lateral E' z-score	0.13 ± 1.11	0.16 ± 0.92	0.29 ± 1.05	0.19 ± 0.85	0.876
Lateral A' z-score	0.18 ± 0.76	0.2 ± 0.87	0.1 ± 0.83	0.14 ± 1.16	0.952
Lateral E/E' z-score	-0.87 ± 1.22	-0.83 ± 1.05	-0.91 ± 0.86	-0.95 ± 0.99	0.942
Septal E' z-score	-0.37 ± 0.82	-0.19 ± 0.77	-0.43 ± 0.9	-0.38 ± 0.66	0.352
Septal A' z-score	-0.34 ± 0.74	-0.41 ± 0.93	-0.4 ± 1.14	-0.42 ± 1.02	0.989
Septal E/E' z-score	-0.67 ± 0.95	-0.69 ± 0.97	-0.46 ± 0.88	-0.65 ± 0.96	0.659
Abnormal inflow pattern	-	3 (4.3)	-	1 (1.9)	-
Pseudo-normal inflow pattern	1 (3.7)	3 (4.1)	-	-	-
Left atrial enlargement	1 (3.7)	4 (5.4)	-	-	-
Diastolic dysfunction signs	2 (7.4)	10 (13.5)	-	1 (1.9)	-

The cohort was divided into four groups based on GDM exposure and maternal obesity (pre-pregnancy BMI ≥ 30 kg/m^2); values are presented as counts or means (± SD); p-value was calculated with one-way ANOVA**.**
GDM – gestational diabetes; **BMI** – body mass index; Significance of the data in bold P < 0.01

Supplemental Table S8. Child diastolic function at six years of age comparison between groups stratified for maternal GDM and child overweight or obesity (mean ± SD or count (%)).

	GDM- / iso-BMI < 25 A	GDM- / iso-BMI ≥ 25 B	GDM+ / iso-BMI < 25 C	GDM+ / iso-BMI ≥ 25 D	ANOVA P-value	B vs A P-value	B vs. C P-value
N	70	32	73	23			
Left atrial volume index (ml/m$^{1.48}$ or ml/m$^{1.08}$) ||	28.5 ± 6.2	28.5 ± 4.9	28.4 ± 5.8	28.0 ± 5.3	0.983		
Left atrial volume index z-score	-0.52 ± 1.1	-0.09 ± 1.11	-0.54 ± 1.05	-0.27 ± 1.12	0.181		
E wave z-score	-1.04 ± 1.03	-0.62 ± 1.12	-0.9 ± 1.08	-0.82 ± 0.89	0.315		
A wave z-score	-0.74 ± 1.25	-0.6 ± 1.14	-0.99 ± 1.13	-0.39 ± 1.25	0.135		
E/A z-score	0.1 ± 1.15	0.24 ± 1.26	0.41 ± 1.12	-0.05 ± 1.14	0.266		
S wave z-score	0.18 ± 0.72	0.38 ± 0.78	0.17 ± 0.78	0.25 ± 0.61	0.584		
D wave z-score	0.14 ± 0.84	-0.3 ± 0.83	-0.07 ± 0.74	0.02 ± 0.67	0.065		
S/D ratio	0.81 ± 0.19	0.9 ± 0.2	0.83 ± 0.17	0.81 ± 0.14	0.141		
Lateral E' z-score	0.17 ± 0.9	0.14 ± 1.12	0.22 ± 0.87	0.26 ± 1.15	0.954		
**Lateral A' z-score**	**-0.01 ± 0.84**	**0.64 ± 0.65**	**0.02 ± 1.02**	**0.4 ± 0.97**	**0.003**	**0.006**	**0.008**
Lateral E/E' z-score	-0.93 ± 1.08	-0.69 ± 1.12	-0.92 ± 0.92	-0.92 ± 0.98	0.717		
Septal E' z-score	-0.22 ± 0.79	-0.32 ± 0.77	-0.39 ± 0.78	-0.54 ± 0.91	0.341		
Septal A' z-score	-0.45 ± 0.88	-0.24 ± 0.87	-0.48 ± 1.08	-0.16 ± 1.0	0.42		
Septal E/E' z-score	-0.79 ± 0.92	-0.44 ± 1.01	-0.54 ± 1.08	-0.43 ± 0.81	0.24		
Abnormal inflow pattern	2 (2.9)	1 (3.1)	1 (1.4)	-	-		
Pseudo-normal inflow pattern	2 (2.9)	2 (6.3)	-	-	-		
Left atrial enlargement	2 (2.9)	3 (9.4)	-	-	-		
Diastolic dysfunction signs	6 (8.6)	6 (18.8)	1 (1.4)	-	-		

The cohort was divided into four groups based on GDM and child overweight or obesity (iso-BMI ≥ 25 kg/m^2); values are presented as counts or means (± SD); P-value was calculated with one-way ANOVA with post-hoc Tukey HSD. **GDM** – gestational diabetes; **BMI** – body mass index, Significance of the data in bold P < 0.01

Supplemental Table S9. Associations of child left ventricle systolic function at six years of age with maternal pre-pregnancy adiposity and gestational glycemia (Pearson correlation coefficient).

	**Pre-pregnancy BMI**	**1st trimester HbA_{1C}**	**3rd trimester HbA_{1C}**	**1st trimester HOMA-IR**	**2nd trimester HOMA-IR**	**3rd trimester HOMA-IR**
Fractional shortening [%]	.102	.023	-.039	.020	-.10	-.031
Ejection fraction [%]	.053	-.128	-.073	-.040	-.138	.002
MAPSE [cm]	.072	.066	-.007	.114	-.020	-.003
Lateral S' [cm/s]	-.035	.058	-.059	.115	.120	.118
Lateral S' Z-score	-.067	.021	-.073	.125	.103	.109
Septal S' [cm/s]	.086	-.078	-.076	.050	-.017	.103
Septal S' Z-score	.075	-.091	-.080	.041	-.024	.099
Longitudinal peak systolic strain [%]	-.093	-.009	-.132	-.076	-.021	-.041
Longitudinal peak systolic strain Z-score	.111	.019	.135	.086	.025	.047
Longitudinal systolic strain rate [1/s]	-.089	-.083	-.201	-.057	-.012	-.030
Basal circumferential peak systolic strain [%]	-.025	.094	.024	.076	.049	.090
Basal circumferential peak systolic strain Z-score	.022	-.097	-.026	-.077	-.051	-.090
Basal circumferential systolic strain rate [1/s]	-.103	.050	.017	-.045	-.091	-.013

BMI – body mass index; **HbA_{1C}** – glycated haemoglobin; **HOMA-IR** – homeostasis model assessment of insulin resistance [fasting insulin (μU/ml) × fasting glucose (mmol/L)/22.5]; † p<0.01, and ‡ p<0.001

Supplemental Table S10. Child left ventricle systolic function associations with body composition, blood pressure, resting heart rate, left ventricle mass, and glucose levels at six years of age (Pearson correlation coefficient).

	**BMI z-score**	**Body fat %**	**Lean body mass**	**SBP z-score**	**DBP z-score**	**Heart rate**	**LVM z-score**	**HbA_{1C}**	**Plasma glucose**	**Insulin**
Fractional shortening [%]	.068	.005	.029	.136	-.040	-.051	-.032	-.067	-.090	-.078
Ejection fraction [%]	.008	.041	-.098	.063	-.130	-.038	.025	-.102	-.088	-.102
MAPSE [cm]	**.241†**	.151	**0.376‡**	-.034	-.151	-.299	.088	-.013	-.061	-.024
Lateral S’ [cm/s]	.055	-.044	**.209†**	-.121	-.067	.131	-.101	-.136	-.073	-.093
Lateral S’ Z-score	-.065	-.116	.035	-.134	-.063	.169	-.109	-.168	-.093	-.134
Septal S’ [cm/s]	.032	.055	-.064	.048	-.009	.176	-.120	-.197	-.127	-.042
Septal S’ Z-score	-.016	.029	-.130	.045	-.005	**.188†**	-.121	**-.206†**	-.133	-.057
Longitudinal peak systolic strain [%]	-.011	-.013	.018	.008	.047	.013	-.149	-.125	.094	.102
Longitudinal peak systolic strain Z-score	.056	.040	.050	-.004	-.045	-.032	1.55	.133	-.085	-.089
Longitudinal systolic strain rate [1/s]	.115	.146	.122	.027	-.058	-.167	-.168	-.193	.022	.095
Basal circumferential peak systolic strain [%]	.013	.019	.10	.066	.142	.129	-.045	.125	.161	.055
Basal circumferential peak systolic strain Z-score	-.025	-.026	-.118	-.066	-.141	-.125	.045	-.128	-.162	-.060
Basal circumferential systolic strain rate [1/s]	.095	.117	.144	.146	-.027	-.214†	.116	.057	.152	.144

BMI – body mass index; **LVM** – left ventricle mass; **SBP** – systolic blood pressure; **DBP** – diastolic blood pressure; **HbA_{1C}** – glycated haemoglobin; **SR** – strain ratio; † p<0.01, and ‡ p<0.001 (two-tailed)

Supplemental Table S11. Child left ventricle systolic function comparison between groups stratified for maternal pre-pregnancy obesity and child overweight or obesity (data are presented as means ± SD or median (IQR)).

	**iso-BMI < 25 / BMI < 30**<br><br>**A**	**iso-BMI < 25 / BMI ≥ 30**<br><br>**B**	**iso-BMI ≥ 25 / BMI <30**<br><br>**C**	**iso-BMI ≥ 25 / BMI ≥ 30**<br><br>**D**	**ANOVA or Kruskal-Wallis P-value**	**D vs A**	**D vs B**
N	55	89	16	39			
Fractional shortening [%]	35.2 ± 2.5	35.7 ± 3.2	35.1 ± 4.2	36.5 ± 3.5	0.25		
Ejection fraction [%]	63.4 ± 3.9	63.7 ± 3.7	62.5 ± 3.5	64.3 ± 4.0	0.46		
MAPSE [cm]	1.45 ± 0.15	1.41 ± 0.15	1.50 ± 0.11	1.54 ± 0.19	**<0.001**	0.03	**<0.001**
Lateral S' [cm/s]	10.4 ± 1.9	10.0 ± 1.9	10.4 ± 1.6	10.3 ± 1.7	0.61		
Lateral S' Z-score	0.40 ± 1.13	0.14 ± 1.07	0.17 ± 0.89	0.08 ± 0.93	0.45		
Septal S' [cm/s]	7.8 ± 0.7	7.7 ± 0.8	7.6 ± 0.8	7.7 ± 0.9	0.85		
Septal S' Z-score	-0.49 (1.15)	-0.52 (1.25)	-1.1 (1.19)	-0.55 (1.23)	0.013		
Longitudinal peak systolic strain [%]	-21.5 ± 1.9	-21.8 ± 2.3	-21.3 ± 2.0	-21.8 ± 2.2	0.73		
Longitudinal peak systolic strain Z-score	0.58 ± 1.10	0.77 ± 1.32	0.54 ± 1.19	0.89 ± 1.27	0.62		
Longitudinal systolic strain rate [1/s]	-1.31 ± 0.14	-1.35 ± 0.15	-1.30 ± 0.16	-1.31 ± 0.15	0.48		
Basal circumferential peak systolic strain [%]	-22.3 (3.6)	-22.5 (3.6)	-22.2 (2.0)	-22.5 (2.0)	0.93		
Basal circumferential peak systolic strain Z-score	1.21 (1.98)	1.33 (2.05)	1.15 (1.13)	1.31 (1.10)	0.92		
Basal circumferential systolic strain rate [1/s]	-1.41 ± 0.13	-1.45 ± 0.17	-1.37 ± 0.11	-1.41 ± 0.17	0.20		

The cohort was divided into four groups based on child overweight or obesity (iso-BMI ≥ 25 kg/m^2) and maternal obesity (pre-pregnancy BMI ≥ 30 kg/m^2); P-value was calculated with one-way ANOVA with post-hoc Tukey's HSD or Kruskal-Wallis test with post-hoc pairwise comparison adjusted by the Bonferroni correction. Significance of the data in bold P < 0.01

Supplemental Table S12. Child left ventricle systolic function comparison between groups stratified for maternal GDM and pre-pregnancy obesity (data are presented as means ± SD or median (IQR)).

	GDM- / BMI < 30<br><br>A	GDM- / BMI ≥ 30<br><br>B	GDM+ / BMI < 30<br><br>C	GDM+ / BMI ≥ 30<br><br>D	ANOVA or Kruskal-Wallis P-value
N	27	74	43	52	
Fractional shortening [%]	35.0 ± 2.8	36.1 ± 3.5	35.1 ± 3.1	35.8 ± 3.2	0.46
Ejection fraction [%]	63.0 ±2.9	63.8 ± 3.9	62.8 ± 4.3	63.8 ± 4.0	0.75
MAPSE [cm]	1.45 ± 0.12	1.46 ± 0.18	1.47 ± 0.14	1.47 ± 0.17	0.94
Lateral S' [cm/s]	10.3 ± 1.7	10.1 ± 1.8	10.2 ± 2.1	10.1 ± 1.9	0.75
Lateral S' Z-score	0.27 ± 1.05	0.11 ± 1.03	0.23 ± 1.20	0.08 ± 1.06	0.58
Septal S' [cm/s]	8.0 (1.0)	8.0 (1.0)	8.0 (1.0)	8.0 (1.0)	0.71
Septal S' Z-score	-0.55 (1.17)	-0.54 (1.28)	-0.49 (1.21)	-0.53 (1.28)	0.61
Longitudinal peak systolic strain [%]	-21.3 ± 1.8	-22.1 ± 2.1	-21.2 ± 2.0	-21.9 ± 2.3	0.36
Longitudinal peak systolic strain Z-score	0.48 ± 1.08	0.95 ± 1.27	0.43 ± 1.13	0.85 ± 1.35	0.31
Longitudinal systolic strain rate [1/s]	-1.30 ± 0.15	-1.34 ± 0.15	-1.31 ± 0.14	-1.32 ± 0.17	0.76
Basal circumferential peak systolic strain [%]	-22.4 ± 1.7	-23.1 ± 2.3	-22.0 ± 2.3	-22.2 ± 1.8	0.046
Basal circumferential peak systolic strain Z-score	1.30 ± 0.96	1.66 ± 1.32	1.08 ± 1.32	1.15 ± 1.03	0.045
Basal circumferential systolic strain rate [1/s]	-1.40 ± 0.13	-1.48 ± 0.18	-1.39 ± 0.13	-1.40 ± 0.15	0.014

The cohort was divided into four groups based on GDM exposure and maternal obesity (pre-pregnancy BMI ≥ 30 kg/m^2); values are presented as counts or means (± SD); P-value was calculated with one-way ANOVA with post-hoc Tukey's HSD or Kruskal-Wallis test with post-hoc pairwise comparison adjusted by the Bonferroni correction. Significance of the data in bold P < 0.01

Supplemental Table S13. Child left ventricle systolic function comparison between groups stratified for maternal GDM and child overweight or obesity (mean ± SD).

	GDM- / iso-BMI < 25<br><br>A	GDM- / iso-BMI ≥ 25<br><br>B	GDM+ / iso-BMI < 25<br><br>C	GDM+ / iso-BMI ≥ 25<br><br>D	ANOVA or Kruskal-Wallis<br><br>P-value	D vs A	D vs C	B vs A	B vs C
N	70	32	73	23					
Fractional shortening [%]	35.3 ± 3.0	36.9 ± 3.8	35.6 ± 2.9	35.2 ± 3.7	0.18				
Ejection fraction [%]	63.1 ± 3.2	64.6 ± 4.1	63.5 ± 4.3	62.9 ± 3.7	0.24				
MAPSE [cm]	1.42 ± 0.16	1.56 ± 0.17	1.46 ± 0.15	1.49 ± 0.18	**0.001**			**<0.001**	0.014
Lateral S’ [cm/s]	9.9 ± 2.0	10.6 ± 1.0	10.2 ± 2.0	10.2 ± 2.1	0.70				
Lateral S’ Z-score	0.12 ± 1.18	0.27 ± 0.59	0.20 ± 1.12	0.03 ± 1.13	0.79				
Septal S’ [cm/s]	8.0 (1.0)	8.0 (1.0)	8.0 (1.0)	7.0 (1.0)	0.173				
Septal S’ Z-score	-0.52 (1.23)	-0.55 (1.48)	-0.49 (1.24)	-1.66 (1.22)	**0.004**	**0.004**	**0.007**		
Longitudinal peak systolic strain [%]	-21.7 ± 2.1	-22.2 ± 1.9	-21.7 ± 2.1	-21.2 ± 2.5	0.44				
Longitudinal peak systolic strain Z-score	0.70 ± 1.24	1.07 ± 1.15	0.71 ± 1.19	0.52 ± 1.45	0.41				
Longitudinal systolic strain rate [1/s]	-1.33 ± 0.15	-1.32 ±0.15	-1.33 ± 0.15	-1.29 ± 0.16	0.62				
Basal circumferential peak systolic strain [%]	-22.9 ± 2.3	-22.8 ± 1.9	-22.2 ± 2.3	-22.0 ± 1.5	0.12				
Basal circumferential peak systolic strain Z-score	1.56 ± 1.31	1.50 ± 1.05	1.15 ± 1.29	1.03 ± 0.82	0.11				
Basal circumferential systolic strain rate [1/s]	-1.46 ± 0.17	-1.42 ± 0.17	-1.40 ± 0.15	1.03 ± 0.82	0.04				

The cohort was divided into four groups based on GDM and child overweight or obesity (iso-BMI ≥ 25 kg/m^2); values are presented as counts or means (± SD); P-value was calculated with one-way ANOVA with post-hoc Tukey’s HSD or Kruskal-Wallis test with post-hoc pairwise comparison adjusted by the Bonferroni correction. Significance of the data in bold P < 0.01

C. Supplemental Figures

Supplemental Fig. S1 Child myocardial relaxation parameters association with adiposity at six years of age:

a) Scatter-plot of child body fat percentage and pulmonary venous flow S/D, with the linear regression line (95% CI).

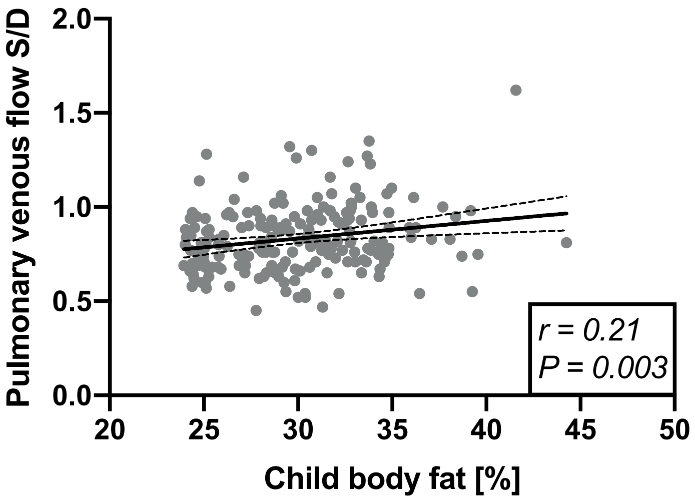

b) Scatter-plot of child body fat percentage and lateral A' Z-score, with the linear regression line (95% CI).

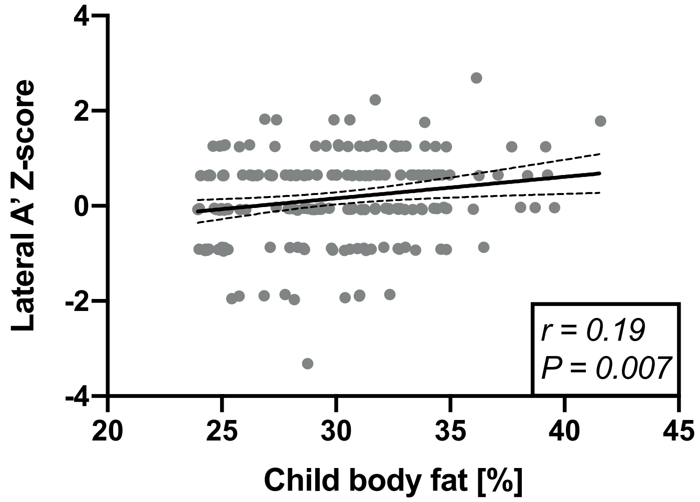

Supplemental Fig. S2 Child left atrial volume association with body composition at six years of age:

a) Scatter-plot of child lean body mass and left atrial volume, with the linear regression line (95% CI).

b) Scatter-plot of child body fat percentage and left atrial volume, with the linear regression line (95% CI).

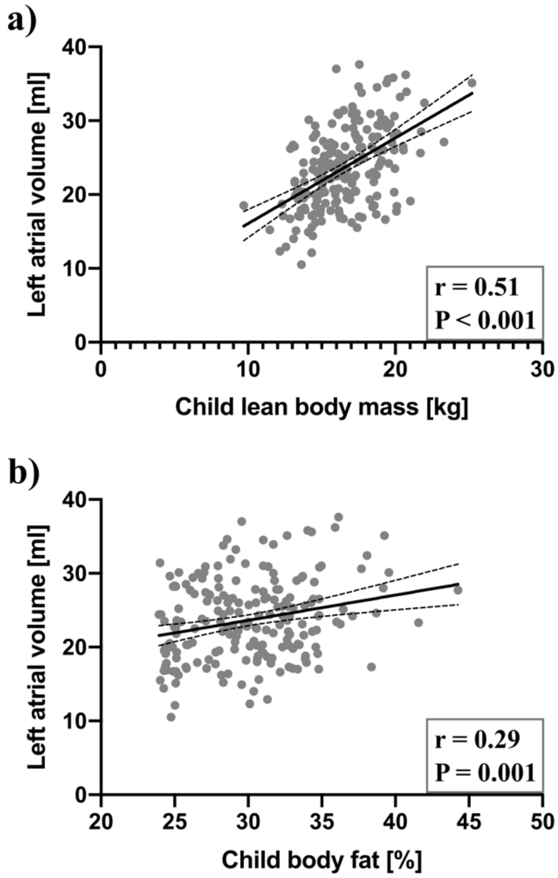

D. References

1. Lee L-W, Liao Y-S, Lu H-K, et al (2017) Validation of two portable bioelectrical impedance analyses for the assessment of body composition in school age children. PLoS One 12:e0171568. https://doi.org/10.1371/journal.pone.0171568

2. Malavolti M, Mussi C, Poli M, et al (2003) Cross-calibration of eight-polar bioelectrical impedance analysis versus dual-energy X-ray absorptiometry for the assessment of total and appendicular body composition in healthy subjects aged 21-82 years. Ann Hum Biol 30:380–391. https://doi.org/10.1080/0301446031000095211

3. Foster BJ, Platt RW, Zemel BS (2012) Development and validation of a predictive equation for lean body mass in children and adolescents. Ann Hum Biol 39:171–182. https://doi.org/10.3109/03014460.2012.681800

4. Bhatla P, Nielsen JC, Ko HH, et al (2012) Normal values of left atrial volume in pediatric age group using a validated allometric model. Circ Cardiovasc Imaging 5:791–796. https://doi.org/10.1161/CIRCIMAGING.112.974428

5. Dallaire F, Slorach C, Hui W, et al (2015) Reference values for pulse wave doppler and tissue doppler imaging in pediatric echocardiography. Circ Cardiovasc Imaging 8:e002167. https://doi.org/10.1161/CIRCIMAGING.114.002167

6. Dallaire F, Slorach C, Bradley T, et al (2016) Pediatric Reference Values and Z Score Equations for Left Ventricular Systolic Strain Measured by Two-Dimensional Speckle-Tracking Echocardiography. J Am Soc Echocardiogr 29:786-793.e8. https://doi.org/10.1016/j.echo.2016.03.018
